# Clinical phenotypes and biologic treatment use in juvenile dermatomyositis-associated calcinosis

**DOI:** 10.1186/s12969-018-0299-9

**Published:** 2018-12-29

**Authors:** Amir B. Orandi, Vikas R. Dharnidharka, Noor Al-Hammadi, Kevin W. Baszis, for the CARRA Legacy Registry Investigators, L. Abramson, E. Anderson, M. Andrew, N. Battle, M. Becker, H. Benham, T. Beukelman, J. Birmingham, P. Blier, A. Brown, H. Brunner, A. Cabrera, D. Canter, D. Carlton, B. Caruso, L. Ceracchio, E. Chalom, J. Chang, P. Charpentier, K. Clark, J. Dean, F. Dedeoglu, B. Feldman, P. Ferguson, M. Fox, K. Francis, M. Gervasini, D. Goldsmith, G. Gorton, B. Gottlieb, T. Graham, T. Griffin, H. Grosbein, S. Guppy, H. Haftel, D. Helfrich, G. Higgins, A. Hillard, J. R. Hollister, J. Hsu, A. Hudgins, C. Hung, A. Huttenlocher, N. Ilowite, A. Imlay, L. Imundo, C. J. Inman, J. Jaqith, R. Jerath, L. Jung, P. Kahn, A. Kapedani, D. Kingsbury, K. Klein, M. Klein-Gitelman, A. Kunkel, S. Lapidus, S. Layburn, T. Lehman, C. Lindsley, M. Macgregor-Hannah, M. Malloy, C. Mawhorter, D. McCurdy, K. Mims, N. Moorthy, D. Morus, E. Muscal, M. Natter, J. Olson, K. O’Neil, K. Onel, M. Orlando, J. Palmquist, M. Phillips, L. Ponder, S. Prahalad, M. Punaro, D. Puplava, S. Quinn, A. Quintero, C. Rabinovich, A. Reed, C. Reed, S. Ringold, M. Riordan, S. Roberson, A. Robinson, J. Rossette, D. Rothman, D. Russo, N. Ruth, K. Schikler, A. Sestak, B. Shaham, Y. Sherman, M. Simmons, N. Singer, S. Spalding, H. Stapp, R. Syed, E. Thomas, K. Torok, D. Trejo, J. Tress, W. Upton, R. Vehe, E. von Scheven, L. Walters, J. Weiss, P. Weiss, N. Welnick, A. White, J. Woo, J. Wootton, A. Yalcindag, C. Zapp, L. Zemel, A. Zhu

**Affiliations:** 1 Division of Pediatric Rheumatology, Department of Pediatric and Adolescent Medicine, Mayo Clinic, 200 First Street SW, Rochester, MN 55902 USA; 2 Division of Pediatric Rheumatology, Department of Pediatrics, St. Louis, MO USA; 3 Division of Pediatric Nephrology, Department of Pediatrics, St. Louis, MO USA; 4 Division of Biostatistics, Washington University School of Medicine, St. Louis, MO USA

**Keywords:** Juvenile dermatomyositis, Calcinosis, Biologics, Pediatric rheumatology

## Abstract

**Background:**

Few risk factors have been identified for the development of calcinosis among patients with Juvenile Dermatomyositis, and currently no clinical phenotype has been associated with its development. We analyzed a large database of patients to further elucidate any relationships among patients with and without calcinosis.

**Method:**

The CARRA legacy registry recruited pediatric rheumatology patients from 55 centers across North America from 2010 through 2014, including over 650 subjects with Juvenile Dermatomyositis. We compared the demographic characteristics, clinical disease features and treatment histories of those with and without calcinosis using univariate and multivariate logistic regression.

**Results:**

Of the 631 patients included in the analysis, 84 (13%) had a current or prior history of calcinosis. These patients were statistically more likely to have longer durations of disease prior to diagnosis and treatment, have lipodystrophy and joint contractures, and to have received intravenous immune globulin or rituximab as treatments.

**Conclusions:**

Calcinosis is found more often in patients with prolonged active disease, severe disease, and certain clinical features such as lipodystrophy and joint contractures. When these factors are combined with other known associations and predictors, groups of at-risk patients can be more effectively identified, treated and studied to improve overall outcomes.

## Introduction

As the most common inflammatory myopathy of childhood, Juvenile Dermatomyositis (JDM) affects 3.2 per million children annually in the United States [1]. Before the widespread use of glucocorticoids, followed by disease-modifying anti-rheumatic drugs (DMARDs), JDM was fatal in one-third of cases [2–4]. More recent studies have shown mortality as low as 3.1% [5] while simultaneously highlighting persistent morbidity from active disease or its sequelae [6–8]. An enigmatic source of morbidity comes from calcinosis, the dystrophic deposition of the mineral calcium hydroxyapatite in the skin, soft tissues or muscle, which is reported to occur in approximately 40% of patients [9]. When surveyed in 2016 regarding their experience and approach to assessing and treating JDM calcinosis, less than 20% of pediatric rheumatologists have treated more than 10 cases. In addition, the majority who have such experience have practiced for more than 20 years. [10].

There have been many studies attempting to identify risk factors or associations of calcinosis in JDM patients in order to further define the at-risk patient population. Actionable risk factors previously identified include a delay to treatment and/or prolonged disease duration [5, 11, 12] and initial treatment intensity [13, 14]; prognostic factors included race [15–17], male sex [15], underlying cardiac disease [18, 19], presence of joint contractures [18], and presence of certain myositis-specific antibodies [20, 21]; genetic risks were also identified [22, 23]. However, in some large cohorts, no risk factors are identified [24]. Due to the rarity of JDM as a whole, and with calcinosis occurring in less than half of patients, there have been few studies of large numbers of patients with calcinosis. Lacking among all of these studies is a clinical phenotype of patient disease features that might also be associated with the development of calcinosis, apart from prolonged or severe disease, which has many connotations depending on the publication. Further, regarding treatment, much attention has been paid to the amount and duration of glucocorticoid use [25–27], but there has been no assessment made of whether the use of biologic treatments, now widely used, are associated with calcinosis. Our aim was to address these questions by analyzing a large patient registry of JDM patients with assessments of clinical features and treatment histories, including those who developed calcinosis during the course of follow-up.

## Methods

The Childhood Arthritis and Rheumatology Research Alliance (CARRA) developed a multicenter registry for pediatric rheumatologic diseases across North America. Patients were enrolled from May 30, 2010 through October 31, 2014 from 55 CARRA centers. JDM was among the diseases included in the registry, and any patients whose disease began before 18 years of age and were less than 21 years of age at the time of enrollment were eligible for inclusion. All JDM patients must have met the Bohan and Peter criteria for diagnosis, modified to allow magnetic resonance imaging as an acceptable diagnostic modality [28].

### Data collection

All data were submitted by the enrolling physician at each site after collecting clinical data using report forms for general information, demographics, functional and quality-of-life measures and JDM-specific information at baseline and follow-up visits. At baseline (the time of registry enrollment), the presence of several disease manifestations was assessed (present, absent or not assessed), including proximal muscle weakness (designated mild, moderate or severe), characteristic disease rashes, lipodystrophy, skin ulceration, periungal telangiectasias and contractures (not specified as due to muscle or joint disease). There was also documentation of presence or absence of other disease features (current, past or never), including calcinosis, small or large joint arthritis, dysphagia or dysphonia and organ involvement (cardiac, gastrointestinal or lung). At follow-up visits, these same clinical features were documented if present or absent. At enrollment, the patient’s history of biologic medication use was assessed by current or prior use, and at follow-up visits, it was assessed if any biologic medication had been used in the visit interval. Patient data were kept in a centralized database by CARRA and after study approval were transmitted as a coded, deidentified dataset under code access agreement to the study team. This study was exempt from the Washington University School of Medicine institutional review board by not constituting human subject research.

### Statistical analyses

All analyses were performed using SAS version 9.4 (SAS Institute Inc) and R version 3.3.1 (R Project for Statistical Computing). Any patients lacking requisite data in calcinosis were excluded. A cross-sectional analysis of baseline data in all JDM patients was performed comparing those with any history (current or past) of calcinosis to those without (never) in respect to demographic characteristics, clinical and disease features, and treatment with biologic agents and glucocorticoids. To calculate the duration of symptoms prior to treatment, the difference between symptom onset date and first rheumatology visit date was used, measured in months. Due to small respective numbers of each form of organ involvement (i.e. cardiac arrhythmia), a composite measure combining all different forms of organ toxicity (i.e. cardiac, pulmonary and gastrointestinal) was also used. For similar reasons, different tumor necrosis factor (TNF)-alpha inhibitors and interleukin-1 inhibitors were grouped as single entities for the purpose of analysis. Information on glucocorticoid treatment was collected in the registry by documenting only current or prior receipt of intravenous pulse steroids and/or systemic daily long-term use of specified durations. No dosages or intervals were recorded. In order to study possible effect of high or low glucocorticoid exposure, selected patients were grouped for the purpose of analysis. A patient with high steroid exposure was defined as one with a current or prior history of intravenous pulse steroids *and* current or prior use of daily systemic corticosteroids of at least one-month duration. Differences between patients with and without calcinosis were analyzed with t-test or Mann-Whitney-U tests for continuous variables as appropriate, while comparisons for categorical variables used Chi-square or Fisher’s exact tests as appropriate. Statistically significant measures at the alpha level of 0.05 on univariate analyses were included in multivariate logistic regression modeling. Stepwise selection method during multivariate logistic regression modeling was used to adjust for collinearity of certain clinical features, where a variable had to be significant at the 0.25 level to be entered into the model and significant at 0.15 to stay. At the following step, the final model included variables significant at 0.05. A Forest Plot using R program was performed to illustrate the odds ratio and associated 95% confidence interval that were significant in the logistic regression model.

## Results

A total of 654 patients with JDM were enrolled in the CARRA legacy registry. Twenty-three patients were excluded who lacked information on calcinosis, including five patients with follow-up data but no information at baseline. The remaining 631 were included in analysis, with 84 patients (13.3%) having a current or prior history of calcinosis at the time of enrollment. The registry cohort included 454 females (72%) and 177 males (28%) with a median age at diagnosis of 5.6 years. Caucasian race comprised 82% of patients, while Hispanic ethnicity represented 15% of patients, and African-American, 13%. The majority of patients were categorized as ‘probable’ JDM, which was used to define 490 patients (78%), while 48 (7.6%) were categorized as definite. Only nine patients (1.4%) were designated as amyopathic, and the remaining 13% had no selected category. Across the entire cohort, 38% had current or prior treatment with a biologic medication (Table 1).

**Table 1:** Cohort characteristics, *n* = 631

Characteristics		
Age (years) at diagnosis (median, IQR)	Median	IQR
	5.6	3.6–9.3
	N	%
Gender		
Male	177	28.1
Female	454	71.9
Ethnicity		
Hispanic	95	15.1
Race		
Caucasian	519	82.3
African-American	82	13
JDM Category		
Amyopathic	9	1.4
Probable	490	77.7
Definite	48	7.6
Missing	84	13.3
History of Calcinosis	84	13.3
History of biologic use	242	38.4
IVIG	225	35.7
Rituximab	26	4.1
Anti-TNF^a^	*35*	*5.6*
Certolizumab	0	0
Etanercept	22	3.5
Golimumab	1	0.2
Adalimumab	7	1.1
Infliximab	13	2
Abatacept	3	0.48
Anti-IL1^b^	2	0.32
High glucocorticoid exposure^c^ (*n* = 572)	316	50

^a^Composite which includes etanercept, infliximab, adalimumab, certolizumab, golimumab

^b^Composite which includes anakinra, canakinumab, rilonacept

^c^Composite defined as history of IV pulse steroids and daily corticosteroids for ≥ 1 month

### Clinical disease features

When comparing those with a history of calcinosis to those without, there were statistically significant differences in the proportions of the two groups for several demographic and clinical features (Table 2). There was no difference between groups in regards to cardiac, lung or gastrointestinal involvement, but when these events were combined as a single composite feature, “any organ involvement”, the differences were statistically significant. In univariate analysis, statistical significance was maintained for characteristics of male sex (OR 1.811, 95% CI 1.123–2.919), time to diagnosis (OR 1.029, 95% CI 1.016–1.043) and African-American race (OR 2.264, 95% CI 1.270–4.038). For other clinical disease features, lipodystrophy (OR 5.993, 95% CI 2.588–13.874), joint contractures (OR 5.343, 95% CI 2.964–9.635), cutaneous ulcerations (OR 3.748, 95% CI 1.787–7.862), Gottron’s or heliotrope rashes (OR 1.754, 95% CI 1.099–2.800), V or shawl sign (OR 2.410, 95% CI 1.083–5.361) and organ involvement (OR 2.506, 95% CI 1.127–5.573) also maintained significance (Table 2) and were included in multivariate analysis.

**Table 2:** Clinical characteristics by history of calcinosis

Risk factor	Descriptive analysis			Univariable analysis	
	Calcinosis			OR [95% CI]	*p*-value
	Yes	No			
Gender	n (%)		0.0138		
Male	33 (39.3)	144 (26.3)		1.811 [1.123–2.919]	0.0148
Female	51 (60.7)	403 (73.7)			
Age (years)	median (IQR)				
At symptom onset	6 (4.2–9)	5.5 (3.6–9.3)			
At diagnosis	8 (5.4–10.6)	6.3 (4.2–9.8)	0.0424		
Time to diagnosis (months)	8.8 (2–23.6)	3.8 (1.9–8.9)	0.0011	1.029 [1.016–1.043]	< 0.0001
Ethnicity	n (%)				
Hispanic	12 (14.3)	83 (15.2)			
Race			0.0047		
Caucasian	61 (72.6)	458 (83.7)			
African-American	19 (22.6)	63 (11.5)		2.264 [1.270–4.038]	0.0056
Missing	4 (4.8)	26 (4.8)			
JDM Category	n (%)				
Amyopathic	2 (2.4)	7 (1.3)			
Probable	61 (72.6)	429 (78.4)			
Definite	8 (9.5)	40 (7.3)			
Missing	13 (15.5)	71 (13)			
Clinical features	n (%)				
Lipodystrophy	11 (13.1)	13 (2.4)	< 0.0001	5.993 [2.588–13.874]	< 0.0001
Joint contractures	23 (27.4)	35 (6.4)	< 0.0001	5.343 [2.964–9.635]	< 0.0001
Cutaneous ulceration	12 (13.5)	23 (4.2)	0.001	3.748 [1.787–7.862]	0.0005
Gottron’s or Heliotrope	50 (59.5)	244 (44.6)	0.0176	1.754 [1.099–2.800]	0.0186
Dysphagia or dysphonia	26 (31)	107 (19.6)	0.0178		
Small joint arthritis	25 (29.8)	115 (21.0)	0.041		
Malar or facial erythema	31 (36.9)	182 (33.3)			
V or Shawl sign	9 (10.7)	25 (4.6)	0.0377	2.410 [1.083–5.361]	0.0311
Periungal Telangiectasia	40 (47.6)	225 (41.1)			
Cardiac involvement	4 (4.8)	10 (1.8)			
Gastrointestinal ulceration	3 (3.6)	7 (1.3)			
Interstitial lung disease	4 (4.8)	11 (2.0)			
Any organ involvement^a^	9 (10.7)	25 (4.6)	0.0333	2.506 [1.127–5.573]	0.0243
Large joint arthritis	26 (31)	147 (26.9)			
Muscle enzyme elevation	74 (88.1)	481 (87.9)			
Severe muscle weakness	2 (2.4)	14 (2.6)			

^a^Composite outcome: includes cardiac, GI or lung involvement

### Treatment with biologics

In the overall cohort, 242 (38.4%) patients experienced current or prior use with a biologic agent. Intravenous immune globulin (IVIG) was the most common with 225 (35.6%) patients exposed followed by anti-TNF-alpha therapy with 35 (5.5%) patients. Etanercept comprised the majority of anti-TNF-alpha therapy with 22 patients reporting use, representing 63% of all anti-TNF-alpha therapy. Rituximab was used in 26 (4.1%) patients. When comparing patients with a history of calcinosis to those without, statistical significance between groups was maintained on univariate analysis for all treatments, except anti-IL1 agents as follows: Rituximab (OR 6.345, 95% CI 2.825–14.245), IVIG (OR 2.353 95% CI 1.479–3.743), anti-TNF-alpha (OR 3.797, 95% CI 1.812–7.960) and abatacept (OR 13.317, 95% CI 1.194–148.514). All were included in multivariable analysis except abatacept, since it was only used in three patients overall, and despite positive odds ratio had an extremely wide confidence interval (Table 3).

**Table 3:** Treatment exposure by history of calcinosis

Risk factor	Descriptive analysis			Univariable analysis	
	Calcinosis			OR [95% CI]	*p*-value
	Yes	No			
Biologic treatment (Ever)	n (%)				
Any biologic	48 (57.1)	194 (35.5)	0.0001	2.426 [1.522–3.867]	
Rituximab	12 (14.3)	14 (2.6)	< 0.0001	6.345 [2.825–14.254]	< 0.001
IV Immune Globulin	45 (53.6)	180 (32.9)	0.0002	2.353 [1.479–3.743]	0.0003
Anti-TNF	12 (14.3)	23 (4.2)	0.0009	3.797 [1.812–7.960]	0.0004
Abatacept	2 (2.4)	1 (0.2)	0.048	13.317 [1.194–148.514]	0.0354
Anti-IL1	1 (1.2)	1 (0.2)			
High steroid exposure	47 (56)	269 (54.3)			

**Fig. 1:**
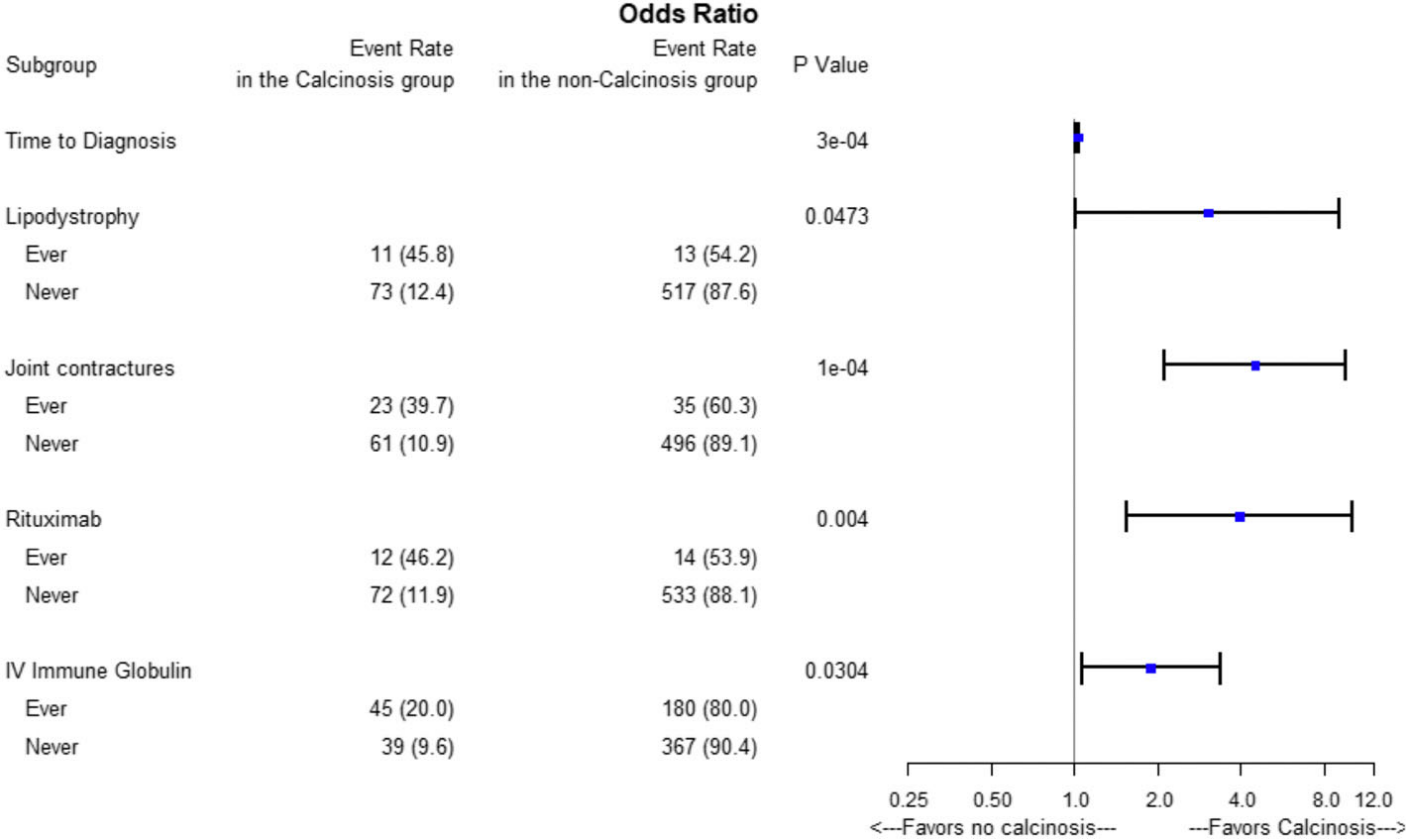
Results of multivariate logistic regression analysis. Risk factors and event rates among patients with and without calcinosis are shown with *p*-values. Forest plot displays odds ratios and associated 95% confidence intervals of multivariate logistic regression

In the multivariable analysis including clinical features and treatments, the only predictors of association with calcinosis were increased time to diagnosis (OR 1.029, 95% CI 1.013–1.045), lipodystrophy (OR 3.038, 95% CI 1.014–9.106), joint contractures (OR 4.499, 95% CI 2.106–9.609), rituximab use (OR 3.955, 95% CI 1.551–10.089), and IVIG use (OR 1.891, 95% CI 1.062–3.366), as shown in Fig. 1.

### Outcomes of calcinosis

Of the 631 patients included in analysis, only 422 had at least one follow-up visit. We reviewed the history of calcinosis in these 422 patients through the first follow-up visit. At baseline enrollment, 356 (84%) patients never had calcinosis and did not develop it through the first follow-up visit. A total of 42 patients had calcinosis present at the time of registry enrollment, and in 11 (26%) of these patients, the calcinosis resolved by the first follow-up visit, while in 31 (74%) patients, the calcinosis persisted. There were 14 patients with a prior history of but no calcinosis present at the time of enrollment. Three of these patients would develop a recurrence by the first follow-up visit.

Ten patients (2.5%) who never had calcinosis at the time of baseline enrollment, developed calcinosis by the first follow-up. The median age of JDM disease onset in these patients was 7 (IQR 4.5–8.2) with a median age at first follow-up (development of calcinosis) of 10.6 years (IQR 9.8–12.4). It should be noted that age of JDM disease onset could be different from age of diagnosis, and age of baseline enrollment. Eight patients were female. Six patients were Caucasian, three patients African-American and one patient mixed Caucasian and Hispanic. At the time of baseline enrollment, one patient had lipodystrophy, while one patient had joint contractures. For patients with available data, most (7/9, 78%) had previously received IV pulse steroids, and 7/9 (78%) were actively receiving daily long-term glucocorticoids for at least 1 month’s duration. Four patients (50%) had previously received IVIG and 4/8 (50%) were actively receiving it. One patient had previously received rituximab (and also previously received IVIG), while one patient was actively receiving rituximab but had never received IVIG. None of the 10 patients had ever received anti-TNF-alpha therapy, abatacept or interleukin-1 inhibitors.

## Discussion

Previous studies have highlighted differences between patients with calcinosis and those without. Duration of active JDM disease has been studied and described in two ways: the duration of symptoms prior to initial diagnosis and treatment, and as chronically active disease despite treatment. These two scenarios are not mutually exclusive. When expressed as the time until diagnosis and treatment, increased symptom duration has been shown to be a risk factor for the development of calcinosis in some studies [5, 11, 18, 29, 30] but not in others [8, 19, 24], including a cohort with a high frequency of calcinosis. When the duration of active disease is expressed as chronically active disease despite treatment (i.e. cumulative disease duration), an association with calcinosis is also found in the studies that examined this feature [5, 6]. The combination of these assessments has led to the understanding that prolonged and/or severe disease activity is a risk factor for calcinosis. This understanding has subsequently been tested, with some evidence that early induction of remission can prevent calcinosis [13, 14]. Myositis antibodies are now widely used to guide prognosis and predict certain complications. In UK cohorts, calcinosis was associated with positive anti-MJ antibodies [20, 21], but this was not replicated in in a North American cohort, in which calcinosis occurred with equal frequency in patients with anti-p155/140, anti-MJ and negative serology [31]. African race, despite a low frequency of patients in all JDM cohorts, has been shown to be a risk factor for calcinosis [15–17]. Previously, the only non-demographic clinical features that were associated with calcinosis were cardiac disease [19] and joint contractures [18].

To our knowledge, our study includes the largest number of JDM patients with calcinosis collected and analyzed as a single cohort. Compared to other international series, our demographic characteristics of sex, age at onset, and JDM classification types are similar [5–8, 16, 24, 29]. We found no association that the age of JDM onset is associated with calcinosis. Male sex was associated with calcinosis on univariate analysis but did not hold significance on multivariable analysis. Our study demonstrated, as others have, that a delay in diagnosis and treatment is associated with the development of calcinosis; however, the effect in our study was small (OR 1.029, 95% CI 1.013–1.045). In addition, we showed that receiving IVIG or rituximab are more strongly associated with ever having calcinosis, and with higher odds ratios (OR 1.891, OR 3.955 respectively) than those with treatment delays. This effect was not seen with our variable of high steroid exposure. Given that IVIG and rituximab are typically reserved for those who fail standard treatment, we suspect those patients had more severe and/or prolonged active disease despite treatment. Clearly, untreated disease represents a risk for developing calcinosis, as was demonstrated in early studies with less effective treatments [2]. A delay in treatment imparts this same risk, and based on the intermittent association of this factor with calcinosis in various studies, this suggests that the effect may possibly be mitigated with *more* effective treatment, in addition to more timely treatment. Perhaps this implies that at even greater risk are the types of disease courses which are chronically active and difficult to control. Many studies, including those focusing on myositis antibodies are attempting to identify the pathophysiological mechanisms underlying these differences and therefore predict which patients might be at risk of calcinosis. If reliable, such predictions would encourage the development of screening techniques and potentially inform treatment approaches. In our study, we found that joint contractures (OR 4.499, 95% CI 2.106–9.609) and lipodystrophy (OR 3.038, 95% CI 1.014–9.106) were both independently associated with developing calcinosis, the latter of which to our knowledge has not been previously reported. Cutaneous ulcerations and any organ involvement (i.e. cardiac, pulmonary or gastrointestinal) were also associated with calcinosis on univariate analyses but did not hold significance in multivariate analysis. These associations may not have been identified in prior studies due to the small numbers of patients with calcinosis as a whole in those studies. Additionally, as collected in the legacy registry, the presence or absence of disease features were assessed simply as current, past or never without reference to chronology. Calcinosis, particularly across a joint may lead to joint contractures [9], which may partially explain this association. Previously, calcinosis was also identified as a predictor for lipodystrophy with affected areas often overlying areas of calcinosis or panniculitis [32]. By reviewing the limited follow-up data in the registry, we also describe that calcinosis occurs infrequently in this cohort over a short time period (2.5% of patients with one follow-up visit), but calcinosis tends to persist or recur once it has developed. It is also noted that in the 10 patients who developed calcinosis, most had received IVIG and/or rituximab suggesting they had either severe or refractory disease, but none of these patients had received anti-TNF-alpha therapy, since monoclonal antibody forms have been shown to be effective against JDM disease activity and calcinosis [33].

The limitations of our study include the retrospective methodology and that patients enrolled in this registry were a convenience sample with varying disease durations at the time of enrollment. Prior histories of treatment and/or complications are subject to recall bias by patients/parents and sufficient documentation at the many respective enrolling centers. Information on calcinosis itself was limited only to its presence or absence without further assessment of phenotype or complications such as infection. There was also no information on whether treatments received by patients were specifically intended for calcinosis or for other JDM disease activity. Additionally, information on testing of myositis-specific and myositis-associated antibodies was not collected. Further, there is inconsistent and relatively few follow-up dates, as the funding for this registry was limited to a short duration of data capture. Given these limitations, however, the CARRA legacy registry has provided important information about pediatric rheumatic diseases and JDM. Many of these limitations have been addressed in the newly created CARRA registry which began enrolling patients in 2015 with plans for several years of prospective follow-up. Apart from the full rationale and methodology already published [34], there are specific improvements in the data capture for JDM and calcinosis that address many of these limitations.

## Conclusion

The pathogenesis and treatment approach for calcinosis remains a frontier of research in Juvenile Dermatomyositis. Identifying patients at risk for the complication is a critical step to improving treatment outcomes by focusing research efforts. From studying a large registry of patients, we confirm prior reports that a delayed time to diagnosis and treatment, joint contractures and organ disease are associated with calcinosis, and we also found that lipodystrophy is similarly associated. More aggressive therapy, namely IVIG and rituximab, in our study cohort is also associated with calcinosis which may reflect chronically active or refractory disease.

## Abbreviations

CARRA: Childhood Arthritis and Rheumatology Research Alliance
CHAQ: Childhood health assessment questionnaire
DMARDs: Disease-modifying anti-rheumatic drugs
IVIG: Intravenous immune globulin
JDM: Juvenile Dermatomyositis
TNF: Tumor necrosis factor
